# Risk Factors, Ultrasonographic-Histopathological Comparison, and Maternal Outcomes of the Placenta Accreta Spectrum: An Ambispective Cohort Study From a Tertiary Care Hospital in Northern India

**DOI:** 10.7759/cureus.115597

**Published:** 2026-09-01

**Authors:** Shivangee Singh, Renu Gupta, Pavika Lal, Vinika Sehgal, Shaily Agarwal, Rashmi Yadav, Ashok K Verma

**Affiliations:** 1 Obstetrics and Gynaecology, Ganesh Shankar Vidyarthi Memorial Medical College, Kanpur, IND; 2 Radiodiagnosis, Ganesh Shankar Vidyarthi Memorial Medical College, Kanpur, IND

**Keywords:** cesarean hysterectomy, maternal morbidity, obstetric hemorrhage, placenta accerta spectrum, placenta previa

## Abstract

Aim: Placenta accreta spectrum (PAS) is one of the leading causes of severe obstetric haemorrhage and peripartum hysterectomy, with a rising incidence due to increasing caesarean delivery rates worldwide. This study aimed to evaluate the risk factors for PAS, assess the concordance between ultrasonographic and histopathological findings, and determine maternal outcomes at a tertiary referral centre in northern India.

Methods: An ambispective observational study was conducted in the Department of Obstetrics and Gynaecology, Upper India Sugar Exchange Maternity Hospital, Ganesh Shankar Vidyarthi Memorial Medical College, Kanpur, India, between January 2020 and July 2024. Retrospective data (January 2020-December 2021) and prospective enrolment (January 2022-July 2024) were included in the study. Women with an antenatal or intraoperative diagnosis of PAS were included. Maternal demographics, obstetric history, intraoperative findings, transfusion requirements, intensive care admission, and maternal morbidity and mortality were recorded.

Results: Seventy women with PAS were identified during the 4.5-year study period. The institutional burden of PAS among women screened based on predefined risk factors increased from 20/720 (2.8%) in the retrospective period to 50/720 (6.9%) during the prospective period, with an overall burden of 70/1,440 (4.9%). Prior caesarean delivery was the dominant associated risk factor (62/70, 88.6%), with two prior caesarean deliveries in 28/70 cases (40.0%). Low-lying placenta was present in 20/70 women (28.6%), while placenta previa coexisted in 26/70 women (37.1%), giving a combined prevalence of 46/70 (65.7%). Caesarean hysterectomy was required in 58/70 women (82.85%), with a mean estimated blood loss of 1854.3 ± 417.3 mL and substantial perioperative transfusion requirements. One maternal death (1/70,1.43%) occurred; 9/70 (12.9%) women required intensive care. Histopathology confirmed all PAS cases (70/70,100%) and served as the reference standard. Ultrasonography correctly identified all cases of placenta increta (17/17, 100%) and placenta percreta (9/9, 100%) but detected only 26/44(59.1%) cases of placenta accreta.

Conclusion: Prior caesarean delivery and coexisting placenta previa were the principal risk factors in this cohort. The rising institutional burden of PAS highlighted the need for early antenatal diagnosis, structured referral pathways, and multidisciplinary perioperative care in tertiary centres.

## Introduction

Placenta accreta spectrum (PAS), formerly known as morbidly adherent placenta, refers to the range of pathological adherence of the placenta, including placenta accreta, placenta increta, and placenta percreta [1]. The most favoured hypothesis regarding the aetiology of PAS is that a defect of the endometrial-myometrial interface leads to a failure of normal decidualisation in the area of a uterine scar, which allows abnormally deep placental anchoring villi and trophoblast infiltration [1].

Over the past four decades, an estimated 5- to 10-fold increase in the incidence of PAS has occurred in developing countries [2]. Caesarean hysterectomy, often performed emergently to control life-threatening haemorrhage, remains the most widely accepted definitive treatment, although uterus-sparing approaches have been described in selected cases [3].

Available data from India describe variable but generally rising institutional rates of PAS, particularly at tertiary referral centres serving populations with high caesarean delivery rates [4,5]. Although ultrasonography is the primary imaging modality for the antenatal diagnosis of PAS, its diagnostic accuracy varies with the depth and extent of placental invasion, and focal placenta accreta may be particularly difficult to identify preoperatively. However, single-centre data from northern India evaluating the combined risk-factor profile, ultrasonographic-histopathological comparison, operative management, transfusion burden, and maternal outcomes of PAS remain limited. Therefore, this study aimed to evaluate the risk factors and maternal outcomes of PAS and to compare its ultrasonographic and histopathological features in women managed at a tertiary referral centre in northern India over a four-and-a-half-year period.

The primary objective of this study was to evaluate the risk factors associated with PAS and to compare ultrasonographic findings with histopathological findings. The secondary objectives were to assess maternal outcomes associated with PAS and to evaluate the relationship between blood loss, transfusion requirements, intensive care unit admission, and other perioperative outcomes.

## Materials and methods

Study design and setting

This ambispective observational study was conducted in the Department of Obstetrics and Gynaecology, Upper India Sugar Exchange Maternity Hospital, in collaboration with the Department of Radiodiagnosis, Ganesh Shankar Vidyarthi Memorial (GSVM) Medical College, Kanpur, Uttar Pradesh, India, over a 4.5-year period from January 2020 to July 2024. The retrospective phase covered January 2020-December 2021, and the prospective phase covered January 2022-July 2024. The institutional protocol for the management of PAS included antenatal ultrasound screening in at-risk women, planned late-preterm caesarean delivery (typically 34-36 weeks), availability of senior obstetricians, anaesthetists, and neonatologists, intraoperative availability of blood products, and intensive care backup.

Study population

Women with clinically suspected PAS, a prenatal diagnosis based on transabdominal ultrasonography, or histopathological confirmation of PAS were eligible. In the prospective arm, cases were identified antenatally using transabdominal ultrasonography, with magnetic resonance imaging performed when indicated. In the retrospective arm, cases were identified from the department's medical records. The final diagnosis and classification of PAS were established using intraoperative findings and histopathological examination according to the FIGO (International Federation of Gynecology and Obstetrics) classification [6].

Patients who met any of the following criteria were included: (i) antenatal diagnosis of PAS on ultrasonography with or without MRI; (ii) clinical suspicion of PAS confirmed intraoperatively; or (iii) retrospective diagnosis of PAS documented in hospital records.

Antenatal women who declined participation during the prospective phase or those with severe pre-existing medical disorders independently affecting maternal outcomes were excluded from the study.

Diagnostic evaluation

All women underwent detailed obstetric transabdominal ultrasonography at our centre. Sonographic features suggestive of PAS included placental lacunae, loss of the retroplacental clear zone, myometrial thinning, interruption of the uterine serosa-bladder interface, and increased subplacental vascularity. MRI was performed selectively in women with inconclusive ultrasonographic findings or when deep placental invasion was suspected.

Variables

Variables recorded included maternal age, parity, number of previous caesarean deliveries, history of uterine surgery (dilatation and curettage, hysteroscopy, and myomectomy), presence of placenta previa on antenatal imaging, gestational age at delivery, mode of delivery, intraoperative findings, estimated blood loss, units of packed red blood cells, fresh frozen plasma, and platelets transfused, requirement for intensive care, length of hospital stay, and maternal mortality.

Statistical analysis

Data were analysed using IBM SPSS Statistics for Windows, Version 26 (Released 2018; IBM Corp., Armonk, New York). Continuous variables were presented as mean ± standard deviation and categorical variables as frequencies and percentages. Pearson correlation analysis was performed to evaluate relationships among selected continuous clinical variables, including estimated blood loss, transfusion requirements, ICU stay, and duration of hospitalisation. A p-value <0.05 was considered statistically significant.

Ethical considerations

The study protocol was approved by the Institutional Ethics Committee of GSVM Medical College, Kanpur, Uttar Pradesh, India (IEC approval number: EC/BMHR/2022/144; date of approval: April 1, 2023). Written informed consent was obtained from all prospectively enrolled participants, and permission for retrospective record review was granted by the same committee. The study was conducted in accordance with the Declaration of Helsinki [7], and patient anonymity and confidentiality were maintained throughout.

## Results

During the four-and-a-half-year study period, 70 women with PAS were identified. Among women screened for PAS based on predefined risk factors, 20/720 (2.8%) were diagnosed with PAS during the retrospective period (January 2020-December 2021), compared with 50/720 (6.9%) during the prospective period (January 2022-July 2024), giving an overall burden of 70/1,440 (4.9%).

Demographic and obstetric characteristics

The mean maternal age was 29.77 ± 3.55 years (range 22-42 years), with most women in the 20-30-year age group. The mean gestational age at delivery was 31.99 ± 5.61 weeks. The distribution of associated risk factors is shown in Table 1. 

**Table 1:** Distribution of associated risk factors among women with placenta accreta spectrum Risk-factor overlap permitted; percentages do not sum to 100. LSCS: lower-segment cesarean section.

Risk factor	n (%)
Previous caesarean delivery (any)	62 (88.6)
One prior LSCS	22 (31.4)
Two prior LSCS	28 (40.0)
Three or more prior LSCS	12 (17.1)
Placenta previa (any grade)	46 (65.7)
Prior dilatation and curettage	11 (15.7)
With previous LSCS	6 (8.6)
Without previous LSCS	5 (7.1)
Prior myomectomy (without previous LSCS)	3 (4.3)
Maternal age ≥35 years	11 (15.7)
Multiparous (≥3 deliveries)	21 (30.0)

Prior surgical history

A history of prior caesarean delivery was present in 62/70 women (88.6%). Among these, 22/70 (31.4%) had one previous caesarean delivery, 28/70 (40.0%) had two previous caesarean deliveries, and 12/70 (17.1%) had three or more previous caesarean deliveries, demonstrating a clear dose-response relationship, with two prior caesarean deliveries representing the most common category. A history of prior dilatation and curettage was documented in 11/70 women (15.7%); among these, 6/11 (54.5%) had a coexisting previous caesarean scar, while 5/11 (45.5%) had no history of caesarean delivery. Low-lying placenta was present in 20/70 (28.6%) women, whereas complete placenta previa coexisted in 26/70 (37.1%) women, as summarised in Table 2.

**Table 2:** Placental location among women with PAS (n = 70) PAS: placenta accreta spectrum.

Placental position	n (%)
Low-lying placenta	20(28.6)
Complete placenta previa	26(37.14)
Other locations	24 (34.28)

Association of placental location with intraoperative diagnosis and antenatal ultrasound findings in women with PAS

Of the 70 cases of PAS, 44/70 (62.9%) were classified as placenta accreta based on intraoperative and histopathological correlation, 17/70 (24.28%) as placenta increta, and 9/70 (12.85%) as placenta percreta. Placental location showed a strong correlation with the severity of PAS on intraoperative and histopathological assessment. Among the 24/70 women (34.3%) with an upper-segment placenta, all 24/24 (100%) had focal placenta accreta intraoperatively; however, antenatal transabdominal ultrasound correctly identified focal accreta in only 8/24 (33.3%) cases. All 18/18 (100%) women with an anterior low-lying placenta were diagnosed with placenta accreta both intraoperatively and on antenatal ultrasound. Similarly, the 2/2 (100%) women with a posterior low-lying placenta had focal accreta confirmed intraoperatively, although neither case was detected antenatally by ultrasound (0/2, 0%). Complete placenta previa was observed in 26/70 women (37.1%), of whom 17/26 (65.4%) had placenta increta and 9/26 (34.6%) had placenta percreta, with complete concordance between antenatal ultrasound and intraoperative diagnosis in both groups, as summarised in Table 3. 

**Table 3:** Association of placental location with intraoperative diagnosis and antenatal ultrasound findings in women with PAS Data are presented as n (%). Percentages in the intraoperative diagnosis column are based on the total study population (N = 70). Percentages in the antenatal USG column represent detection within each subgroup. PAS, placenta accreta spectrum; USG, ultrasonography.

Placental location	Type of placental attachment	Intraoperative diagnosis, n (%)	Antenatal USG diagnosis, n (%)
Upper segment	Focal accreta	24 (34.3)	8 (33.3% of subgroup)
Low-lying anterior	Placenta accreta	18 (25.7)	18 (100% of subgroup)
Low-lying posterior	Focal accreta	2 (2.9)	0 (0% of subgroup)
Complete placenta previa (increta)	Placenta increta	17 (24.3)	17 (100% of subgroup)
Complete placenta previa (percreta)	Placenta percreta	9 (12.9)	9 (100% of subgroup)

Comparison of antenatal ultrasonography with histopathological diagnosis

Histopathological examination was considered the reference standard for the final diagnosis of placenta accreta spectrum. Among 70 confirmed cases, histopathology demonstrated 44/70 (62.9%) cases of placenta accreta, 17/70 (24.3%) cases of placenta increta, and 9/70 (12.9%) cases of placenta percreta. Antenatal ultrasonography correctly identified all cases of placenta increta (17/17, 100%) and placenta percreta (9/9, 100%); however, 2D transabdominal ultrasonography detected only 26/44 (59.1%) histopathologically confirmed cases of placenta accreta, underestimating the burden of focal placenta accreta. These findings highlight the higher detection of deeper placental invasion by ultrasonography while demonstrating lower detection of focal placenta accreta. The comparison is summarised in Table 4. 

**Table 4:** Comparison of antenatal ultrasonography and histopathological diagnosis of placenta accreta spectrum Histopathology confirmed all placenta accreta spectrum cases and served as the reference standard. Ultrasonography correctly identified all cases of placenta increta and placenta percreta but underestimated placenta accreta.

PAS subtype	Ultrasonography, n (%)	Histopathology, n (%)
Placenta accreta	26 (37.1%)	44 (62.9%)
Placenta increta	17 (24.3%)	17 (24.3%)
Placenta percreta	9 (12.9%)	9 (12.9%)

Operative outcomes

The mean estimated blood loss was 1854.3 ± 417.3 mL. Overall, 48/70 (68.6%) women required transfusion of ≥2 units of packed red blood cells. Fresh frozen plasma and platelet concentrates were each administered to 41/70 (58.6%) women. In women requiring massive transfusion, blood components were administered according to a 1:1:1 massive transfusion protocol, comprising packed red blood cells, fresh frozen plasma, and platelet concentrates. Operative outcomes are summarised in Table 5.

**Table 5:** Operative outcomes and postoperative course (n = 70) PRBC: packed red blood cells; FFP: fresh frozen plasma; ICU: intensive care unit; SD: standard deviation.

Variable	n (%)	Mean ± SD
Estimated blood loss (mL)	–	1854.3 ± 417.3
PRBC transfused (units)	–	1.94 ± 1.27
FFP transfused (units)	–	1.43 ± 1.36
Platelet concentrates transfused (units)	–	1.44 ± 1.25
Intraoperative cystotomy/urological involvement	4 (5.7)	–
Adnexal involvement	3 (4.3)	–
Cesarean hysterectomy	58 (82.85)	–
Conservative uterine preservation	12 (17.14)	–
Relaparotomy/re-exploration	5 (7.1)	–
ICU admission	9 (12.9)	–
ICU stay, days (among those admitted)	–	1.46 ± 1.62
Total hospital stay, days	–	6.91 ± 2.71
Maternal death	1 (1.43)	–

Maternal morbidity and mortality

Planned caesarean hysterectomy with the placenta left in situ was performed in 58/70 (82.85%) women. The mean length of hospital stay was 6.91 ± 2.71 days; 9/70 (12.9%) women required intensive care for a mean of 1.46 ± 1.62 days. One maternal death occurred (1/70, 1.43%): the patient was a 34-year-old gravida 4 with two prior caesarean deliveries in whom intractable postpartum haemorrhage necessitated emergency hysterectomy, followed by dilutional coagulopathy, multiorgan dysfunction, and septicaemia, leading to death on postoperative day 5 despite aggressive resuscitation and intensive care. No other direct or indirect maternal deaths occurred.

Correlation analysis

Pearson correlation analysis demonstrated strong positive correlations between estimated blood loss and transfusion of packed red blood cells (r = 0.685, p < 0.0001), fresh frozen plasma (r = 0.700, p < 0.0001), and platelet concentrates (r = 0.685, p < 0.0001). Greater blood loss was also associated with prolonged ICU stay (r = 0.663, p < 0.0001) and longer total hospital stay (r = 0.458, p < 0.0001). Preoperative haemoglobin showed significant negative correlations with PRBC transfusion (r = −0.643, p < 0.0001) and blood loss (r = −0.369, p = 0.0017), indicating that lower haemoglobin levels were associated with increased perioperative blood loss and transfusion requirements. Gestational age demonstrated moderate negative correlations with transfusion requirements, whereas maternal age showed no statistically significant association with blood loss. The overall relationships among study variables are illustrated in Figure 1 and Table 6. 

**Table 6:** Clinically relevant Pearson correlations among study variables Correlation coefficients and p-values are summarised from the complete Pearson correlation matrix. PRBC: Packed red blood cells; FFP: Fresh frozen plasma; ICU: Intensive care unit.

Variables compared	Pearson correlation coefficient (r)	p-value	Interpretation
Blood loss vs PRBC transfusion	0.685	<0.0001	Strong positive correlation
Blood loss vs FFP transfusion	0.700	<0.0001	Strong positive correlation
Blood loss vs platelet transfusion	0.685	<0.0001	Strong positive correlation
Blood loss vs ICU stay	0.663	<0.0001	Strong positive correlation
Blood loss vs total hospital stay	0.458	<0.0001	Moderate positive correlation
Haemoglobin vs PRBC transfusion	−0.643	<0.0001	Strong negative correlation
Haemoglobin vs blood loss	−0.369	0.0017	Moderate negative correlation
Gestational age vs PRBC transfusion	−0.548	<0.0001	Moderate negative correlation
Gestational age vs FFP transfusion	−0.447	0.0001	Moderate negative correlation
Maternal age vs blood loss	−0.199	0.0985	No statistically significant correlation

**Figure 1:**
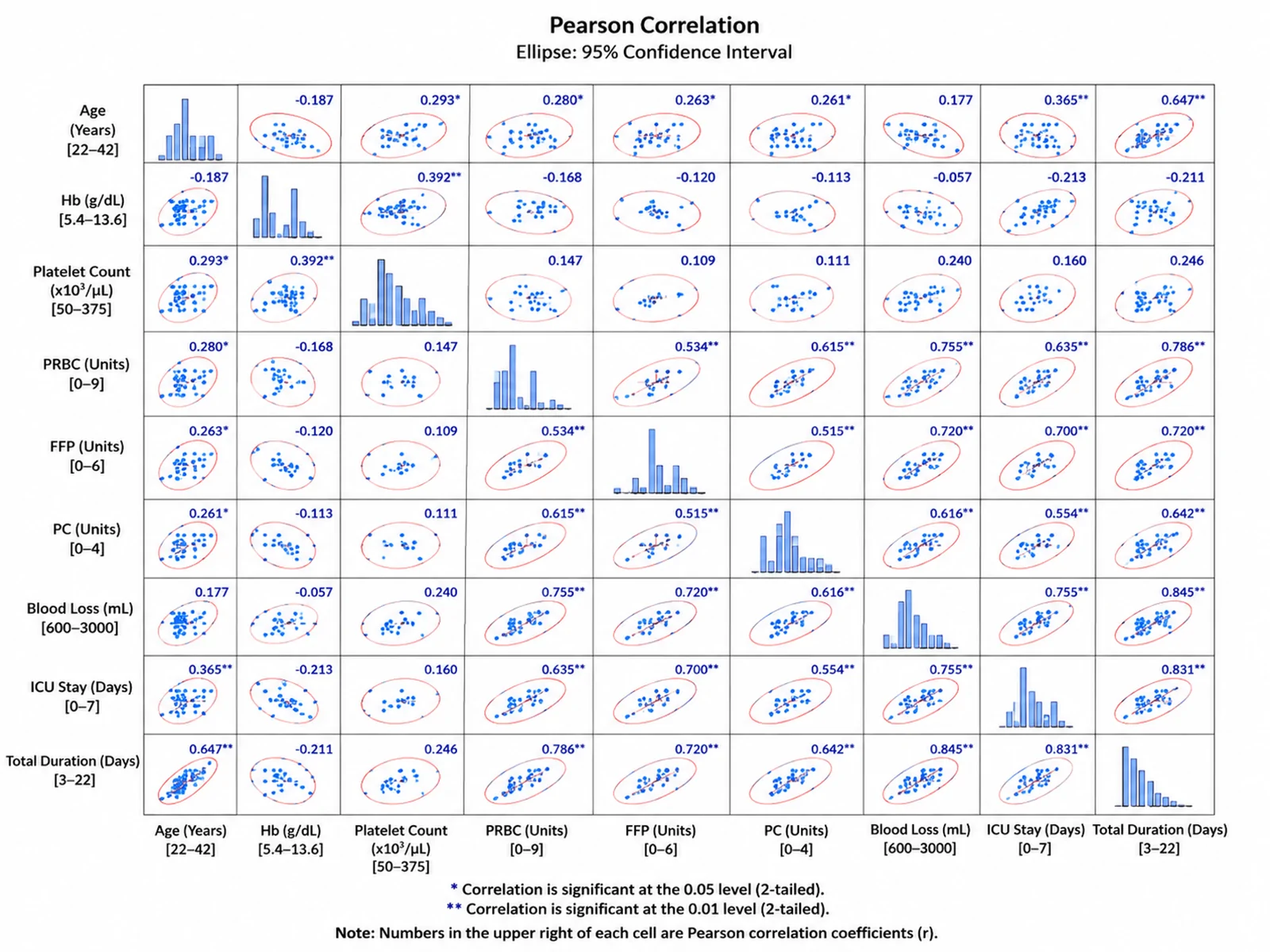
Pearson correlation ellipse plot demonstrating relationships among demographic, hematological, operative, transfusion, and postoperative variables in women with placenta accreta spectrum The correlation ellipse plot was generated using OriginPro (OriginLab Corporation, Northampton, Massachusetts). PRBC: packed red blood cells; FFP: fresh frozen plasma; PC: platelets; ICU: intensive care unit.

## Discussion

The present ambispective cohort of 70 women with PAS, managed at a single tertiary referral centre catering to Kanpur and adjoining areas in Uttar Pradesh, India, over a four-and-a-half-year period, adds to the descriptive regional literature on the morbidity pattern of this condition. Among women screened for PAS based on predefined risk factors, 20/720 (2.8%) were diagnosed with PAS during the retrospective period, compared with 50/720 (6.9%) during the prospective period, giving an overall burden of 70/1,440 (4.9%). Hospital-level rates reflect referral patterns and antenatal detection in addition to true population incidence, but the direction of change mirrors the upward trajectory of PAS documented internationally over the past two decades [2,8]. Contemporary FIGO documents attribute this increase principally to the growing number of women entering pregnancy with a scarred uterus [9].

Prior caesarean delivery emerged as the dominant associated factor in the present cohort. Overall, 62/70 women (88.6%) had a history of one or more previous caesarean deliveries, with two prior caesarean deliveries being the most frequent category (28/70, 40.0%). This observation is consistent with the well-established association between previous caesarean delivery and the risk of PAS, which increases progressively with the number of prior caesarean deliveries [10]. The predominance of women with two previous caesarean deliveries in our cohort likely reflects regional obstetric practices, where a substantial proportion of women undergo permanent sterilisation following a second caesarean delivery, thereby limiting subsequent pregnancies after three or more caesarean deliveries. Consequently, the higher absolute number of PAS cases among women with two previous caesarean deliveries in the present study should not be interpreted as indicating a greater risk than that associated with three or more prior caesarean deliveries. From a population health standpoint, the present findings support the WHO priority to reduce unnecessary primary caesarean deliveries as a means of mitigating downstream obstetric morbidity and strengthen the case for careful institutional audit of primary caesarean indications [11].

Placenta previa coexisted in 46/70 women (65.7%), supporting its established role as a key cofactor. The combination of placenta previa in a scarred uterus corresponds to the clinical scenario repeatedly identified in both Green-top Guideline and ACOG documents as conferring the greatest individual risk of PAS [3,12]. The mechanistic basis applies directly to the present cohort: defective decidualisation at the site of a healed lower-segment scar plausibly permits abnormal trophoblastic invasion. In practical terms, any woman with a low-lying placenta on antenatal imaging and at least one prior caesarean delivery should be presumed high-risk and referred for specialist evaluation. Notably, prior dilatation and curettage was present in 11/70 women (15.7%); of these, 6/70 women (8.6%) had a coexisting previous caesarean scar and 5/70 (7.1%) women did not, suggesting that endometrial trauma from any source may contribute to abnormal placental adherence [8,13].

Placenta accreta accounted for 44/70 (62.9%) of cases, increta for 17/70 (24.3%), and percreta for 9/70 (12.9%) cases, a distribution similar to that reported in population-level analyses, in which accreta remains the most common subtype [14]. Although fewer than one in eight women had percreta, this subgroup contributed disproportionately to caesarean hysterectomy and transfusion burden, consistent with the broader observation that depth of invasion, rather than absolute subtype frequency, is the principal determinant of operative difficulty [8,15].

Caesarean hysterectomy was performed in 58/70 (82.9%) women, in keeping with current consensus guidance endorsing planned hysterectomy with the placenta left in situ as the preferred definitive procedure where future fertility is not a priority [12]. Conservative approaches preserving the uterus were reserved for selected women after detailed counselling and remain an area of active evaluation in well-equipped centres [16]. The mean estimated intraoperative blood loss of 1854.3 ± 417.3 mL, together with substantial transfusion requirements, underscores the significant haemorrhagic burden associated with PAS. In women requiring massive transfusion, blood components were administered in a 1:1:1 ratio of packed red blood cells, fresh frozen plasma, and platelet concentrates, in accordance with institutional practice to facilitate balanced component therapy and reduce the risk of dilutional coagulopathy [17]. ICU admission in 9/70 (12.9%) women and a mean total hospital stay approaching 7 days further underscore the resource intensity of multidisciplinary management. The correlation analysis reinforces the clinical importance of haemorrhage in placenta accreta spectrum [18]. Increasing intraoperative blood loss was strongly associated with greater blood component transfusion, prolonged ICU stay, and longer hospitalisation. In addition, lower preoperative haemoglobin levels were associated with higher transfusion requirements, emphasising the value of optimising maternal haematological status before planned delivery in women at risk of PAS [19]. One maternal death (1/70, 1.43%) occurred in a 34-year-old gravida with two prior caesarean deliveries in whom progressive refractory postpartum haemorrhage, coagulopathy, and multiorgan dysfunction developed despite aggressive resuscitation, exemplifying how even a prepared multidisciplinary pathway may fail to avert the worst outcomes in PAS.

Antenatal diagnosis in our cohort relied principally on expert obstetric ultrasonography performed by experienced sonologists, with selective use of MRI when ultrasonographic findings were equivocal or deep invasion was suspected. Histopathological examination confirmed all cases of placenta accreta spectrum and served as the diagnostic reference standard. While antenatal ultrasonography demonstrated excellent agreement with histopathology in diagnosing placenta increta and placenta percreta, it detected only 26/44 (59.1%) histopathologically confirmed cases of placenta accreta. This finding suggests that superficial placental adherence remains more difficult to diagnose antenatally than deeper myometrial invasion. Factors such as subtle sonographic findings, operator dependency, placental location, and variable myometrial thinning may contribute to underdiagnosis of placenta accreta [20]. Nevertheless, ultrasonography remains the primary imaging modality for antenatal screening because accurate identification of increta and percreta enables timely multidisciplinary planning and reduces maternal morbidity.

Strengths and limitations

The consecutive inclusion of cases over a sustained 4.5-year period, the consistency of the institutional protocol between the retrospective and prospective periods, the multiple categories of variables captured (risk factors, intraoperative findings, transfusion requirements, and outcomes), and histopathological corroboration together constitute the principal strengths of this study. Limitations include the single-centre, descriptive design, with attendant referral bias inflating hospital-based rates above true population incidence; the modest sample size, which precludes meaningful subgroup analyses and multivariable adjustment; the absence of a contemporaneous non-PAS comparator group within the same institution; the lack of standardised cost analysis; and incomplete capture of neonatal outcomes (birthweight, Apgar scores, and NICU admission). Findings should therefore be interpreted as institution-specific and not generalised to obstetric populations with differing case mixes or service configurations. Further multicentre studies are needed to characterise the burden and outcomes of PAS across different regions of India and to strengthen locally appropriate management pathways.

## Conclusions

Placenta accreta spectrum remains a major cause of maternal morbidity in tertiary obstetric practice, particularly among women with previous caesarean delivery and coexisting low-lying placenta or placenta previa. Early recognition of high-risk pregnancies, structured antenatal imaging, timely referral, and multidisciplinary preparation are essential to reduce preventable maternal morbidity. Management should be individualised according to the extent of placental invasion, maternal condition, institutional expertise, and reproductive preferences. Adequate blood bank preparedness and coordinated perioperative care are particularly important because of the potential for severe haemorrhage.
